# Characterization of *Salmonella* Phage P1-CTX and the Potential Mechanism Underlying the Acquisition of the *bla*_CTX-M-27_ Gene

**DOI:** 10.3390/antibiotics13050446

**Published:** 2024-05-14

**Authors:** Qiu-Yun Zhao, Run-Mao Cai, Ping Cai, Lin Zhang, Hong-Xia Jiang, Zhen-Ling Zeng

**Affiliations:** 1 Guangdong Key Laboratory for Veterinary Pharmaceutics Development and Safety Evaluation, College of Veterinary Medicine, South China Agricultural University, Guangzhou 510642, China; qyzhao@zafu.edu.cn (Q.-Y.Z.); crm1995my@163.com (R.-M.C.); molvecc@163.com (P.C.); zhanglin9872@163.com (L.Z.); hxjiang@scau.edu.cn (H.-X.J.); 2 Key Laboratory of Applied Technology on Green-Eco-Healthy Animal Husbandry of Zhejiang Province, Zhejiang Provincial Engineering Laboratory for Animal Health Inspection & Internet Technology, Zhejiang International Science and Technology Cooperation Base for Veterinary Medicine and Health Management, China-Australia Joint Laboratory for Animal Health Big Data Analytics, College of Animal Science and Technology & College of Veterinary Medicine of Zhejiang A&F University, Hangzhou 311300, China

**Keywords:** P1-CTX phage, *Salmonella*, *bla*_CTX-M-27_, acquisition mechanism

## Abstract

The P1 phage has garnered attention as a carrier of antibiotic resistance genes (ARGs) in Enterobacteriaceae. However, the transferability of ARGs by P1-like phages carrying ARGs, in addition to the mechanism underlying ARG acquisition, remain largely unknown. In this study, we elucidated the biological characteristics, the induction and transmission abilities, and the acquisition mechanism of the *bla*_CTX-M-27_ gene in the P1 phage. The P1-CTX phage exhibited distinct lytic plaques and possessed a complete head and tail structure. Additionally, the P1-CTX phage was induced successfully under various conditions, including UV exposure, heat treatment at 42 °C, and subinhibitory concentrations (sub-MICs) of antibiotics. Moreover, the P1-CTX phage could mobilize the *bla*_CTX-M-27_ gene into three strains of *Escherichia coli* (*E. coli*) and the following seven different serotypes of *Salmonella*: Rissen, Derby, Kentucky, Typhimurium, Cerro, Senftenberg, and Muenster. The mechanism underlying ARG acquisition by the P1-CTX phage involved Tn*1721* transposition-mediated movement of *bla*_CTX-M-27_ into the *ref* and *mat* genes within its genome. To our knowledge, this is the first report documenting the dynamic processes of ARG acquisition by a phage. Furthermore, this study enriches the research on the mechanism underlying the phage acquisition of drug resistance genes and provides a basis for determining the risk of drug resistance during phage transmission.

## 1. Introduction

In recent years, the rapid dissemination of antibiotic resistance genes (ARGs) among bacteria has posed significant challenges to the treatment of bacterial infections [1]. CTX-M-type extended spectrum β-lactamases (ESBLs) are major genetic determinants that confer bacterial resistance to cephalosporins. Since the CTX-M enzyme was discovered in German *E. coli* in 1990, at least 220 CTX-M subtypes have subsequently been found. In recent years, the prevalence of the CTX-M-27 subtype has increased in human *E. coli* strains across Europe, North America, Japan, South Korea, and China [2,3,4,5,6,7,8]. The emergence and spread of CTX-M-27-positive *E. coli* and *Salmonella* have also been reported in humans and animals in China [8,9]. As the presence of CTX-M-27-positive strains continues to increase in both humans and animals, there is a growing interest in determining the mechanisms underlying the emergence and transmission of the *bla*_CTX-M-27_ gene.

Mobile genetic elements (MGEs), such as insertion sequences, transposons, and plasmids, frequently facilitate ARG transfer among clinical isolates [10]. Prophages constitute approximately 20% of bacterial genomes and play a crucial role in mediating gene exchange between bacteria at a high frequency through phage transduction-mediated horizontal gene transfer events [11]. This role enables bacteria to rapidly adapt to environmental challenges during evolution and contributes to virulence and pathogenesis [12]. The P1 phage is an independent circular plasmid with a low copy number within several enteric bacteria species that is particularly valuable for molecular biology studies. Recently, the P1 phage has attracted increasing attention as a carrier of ARGs within Enterobacteriaceae family members. Researchers have documented that the P1 phage can harbor *bla*_CTX-M-27_, *mcr-1*, *bla*_SHV-2_, or *bla*_CTX-M-55_ genes as part of a phage−plasmid complex or when fused with other replicon-type plasmids [13,14,15,16,17].

Our recent research revealed, for the first time, that a P1 phage carrying an 8.6-kb Tn*1721*-*bla*_CTX-M-27_ sequence (referred to as the P1-CTX phage) mediated high-level resistance to third generation cephalosporins (3GCs) in a *Salmonella* J46 strain isolated from a slaughterhouse [16,17]. Additionally, further investigation revealed that P1-like phages found in *Salmonella* and different *E. coli* replicon-type plasmids present in livestock animals contained Tn*1721*-like structures with significant genetic similarities [17], suggesting that *bla*_CTX-M-27_ mobilized through a Tn*1721*-like structure between plasmids of *Salmonella* and *E. coli*. However, it remains largely unknown whether P1-like phages carrying ARGs in different hosts can transfer ARGs through transduction or how they can acquire ARGs. This study aimed to evaluate the biological characteristics, the induction and transmission ability, and the acquisition mechanism of ARGs by the P1-CTX phage.

## 2. Results and Discussion

### 2.1. Plaque and Morphology of the P1-CTX Phage

**Figure 1:**
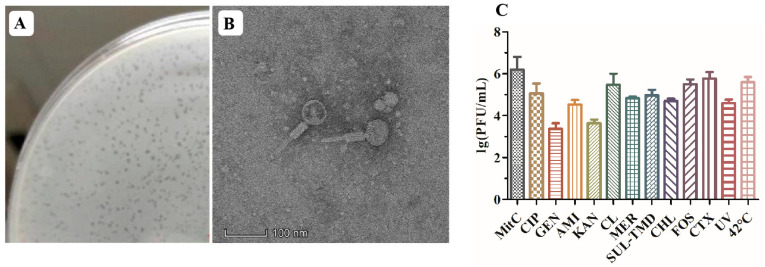
P1-CTX phage activity. (**A**): P1-CTX phage plaque; (**B**): transmission electron microscopy image of P1-CTX phage; (**C**): induction efficiency under different conditions. MitC: mitomycin C; CIP: ciprofloxacin; GEN: gentamicin; AMI: amikacin; KAN: kanamycin; CL: colistin; MER: meropenem; SUL-TMD: sulfamethoxazole-trimethoprim; CHL: chloramphenicol; FOS: fosfomycin; CTX: cefotaxime; UV: ultraviolet.

The induction of the P1-CTX phage in *Salmonella* J46 by mitomycin C (MitC) resulted in the formation of clear plaques on a double-layer agar plate that featured diameters ranging from 1 to 2 mm (Figure 1A). Although two intact phages (P1-CTX and Salmon_SEN8_NC_047753) were predicted to be present in *Salmonella* J46, the plaques on BYC4 were uniform in size and were validated to be P1-CTX phage plaques. Further characterization through transmission electron microscopy (TEM) images revealed that the P1-CTX phage exhibited an icosahedral structure with DNA-containing heads that were approximately 80.0 ± 5.0 nm in length between opposite apices. The length of the tail was approximately 140.0 ± 10.0 nm (Figure 1B). The P1-CTX phage has the same morphology and size as the previously reported P1 phage [18]. These findings provide valuable insights into the morphology and dimensions of the P1-CTX phage present in *Salmonella* J46 under induced conditions. The clear plaques formed on the agar plate indicate that the bacteriophage successfully replicated and exhibited lytic activity against its host bacteria.

### 2.2. Induction Conditions for the P1-CTX Phage

After exposure to sub-MIC levels of the tested antibiotics at 42 °C and under UV radiation, lysogen J46 generated the lysable P1-CTX phage (Figure 1C). Notably, each antibiotic, UV radiation exposure, and 42 °C temperature had an impact on the abundance of phage particles produced. Temperature played a significant role in inducing phage generation; low temperatures (37 °C) inhibited P1-CTX phage generation, and high temperatures facilitated efficient spread of the *bla*_CTX-M-27_ gene (at 42 °C). Among all tested antibiotics, sub-MIC levels of cefotaxime and fosfomycin induced the highest rates for the *bla*_CTX-M-27_ gene because their mechanisms of action were different than those of aminoglycosides (such as amikacin, gentamycin, and kanamycin); however, all antibiotics tested induced the P1-CTX phage harboring the *bla*_CTX-M-27_ gene.

Previous studies have shown that exposure to sub-MIC levels of certain antibiotics, such as β-lactam, trimethoprim and ciprofloxacin, can enhance phage induction in vitro and increase the ability of virulence genes to transfer between laboratory strains [19,20]. When lysogens are exposed to antibiotics under specific conditions, such as those mentioned above, they may promote genetic exchange within bacterial populations, leading to bacterial evolution and resulting in improved fitness.

### 2.3. Transduction of the P1-CTX Phage

The ability of the P1-CTX phage to transduce different *Salmonella* and *E. coli* strains was tested. Transductants carrying the P1-CTX phage were identified in 24 out of 200 *Salmonella* strains and 3 out of 200 *E. coli* strains. The *Salmonella* serotypes harboring P1-CTX transductants included *S.* Rissen (*n* = 5), *S.* Derby (*n* = 5), *S.* Kentucky (*n* = 5), *S.* Typhimurium (*n* = 4), *S.* Cerro (*n* = 3), *S.* Senftenberg, and *S*. Muenster (Table 1). The transduction frequencies of the P1-CTX phage ranged from 1.1× 10^−5^ to 7.9 × 10^−3^ transductants per recipient cell at a multiplicity of infection of 1.

Although numerous antibiotic resistance genes (ARGs) were found in P1 phages, such as *bla*_CTX-M-55_, *bla*_CTX-M-15_, *bla*_KPC-2_, *mcr-1*, and *bla*_SHV-2_, only two studies have reported the production and transduction of P1 phages carrying ARGs [13,21,22,23,24]. In Wang’s study, a P1 phage carrying *bla*_CTX-M-55_ was induced in duck-derived *E. coli* but lost its lytic potential [13]. Another study demonstrated that the P1 phage carried multiple resistant regions and could be transduced into *E. coli* [14]. To date, information on P1 phages reported in *Salmonella* has been limited to *S.* Typhimurium and *S.* Choleraesuis strains, with most reports focusing on the presence of P1 phages in *E. coli* [25]. In contrast to previously reported P1 phages, the P1-CTX phage described here exhibited transduction ability toward *E. coli* and showed transduction capability across seven different serotypes, particularly *S*. Derby, *S*. Rissen, *S*. Kentucky, *S*. Typhimurium, and *S*. Cerro.

### 2.4. Movement Mechanism of Tn Acquisition by P1 Phage

**Figure 2:**
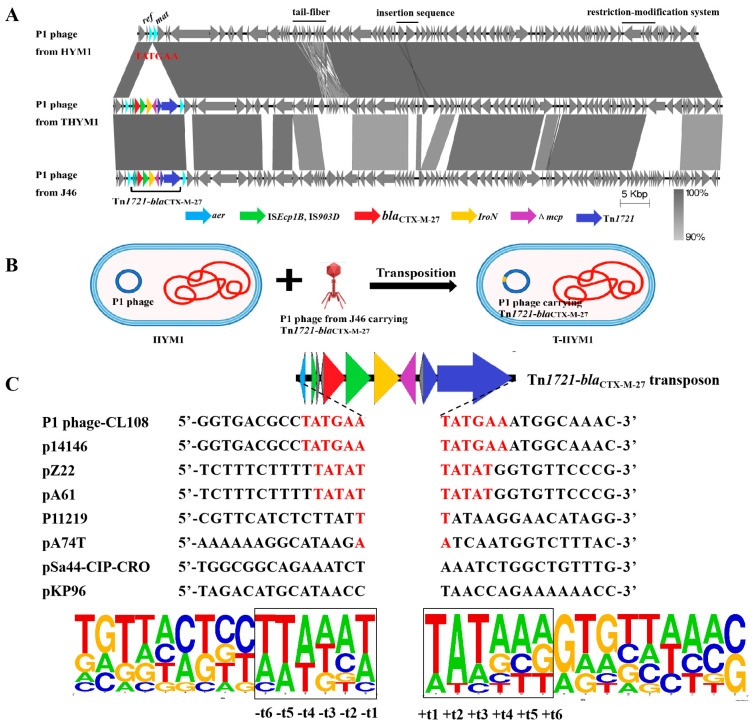
Target site analyses of Tn*1721*-*bla*_CTX-M-27_ transposons. (**A**): Sequence comparison of P1-CTX phage and P1 phage in HYM1 and T-HYM1; (**B**): transposition diagram of transposon Tn*1721*-*bla*_CTX-M-27_ between P1 phages; (**C**): target site analyses of Tn*1721* transposons.

Among the 27 transductants tested, the presence of the P1-CTX phage was detected in 26 transductants (excluding transductant *E. coli* HYMl named T-HYM1). The P1 phage harboring *bla*_CTX-M-27_ found in T-HYMl (named the p1-T-HYMl phage) shared approximately 60% similarity with the SJ46 strain-specific P1-CTX phage. Additionally, differences were observed between these two strains regarding their restriction−modification genes, tail fiber-related genes, and morphogenetic function genes. However, a high sequence identity (99%) was observed between the p1-T-HYM1 phage and p1-HYM1 phage (the p1-HYM1 phage is the original phage in HYM1) (Figure 2A), with only one Tn*1721*-*bla*_CTX-M-27_ transposon inserted between the *ref* and *mat* genes in pl-HYM1 (CP141766). Mobilization of the Tn*1721*-*bla*_CTX-M-27_ transposon from p1-T-HYM1 to the *ref* and *mat* gene loci of p1-HYM1 likely occurred through the action of the Tn*1721* transposon (Figure 2B).

Further investigation into the insertion sites of Tn*1721*-*bla*_CTX-M-27_ transposons revealed varying sequences in different plasmids or P1 phages from the NCBI data. The target site duplication (TSD) sequence of the P1-CTX phage and plasmid p14146 (CP064673) was a 6-bp sequence (TATGAA) [26], while plasmids pZ22 and pA74T (MT587865 and MG014720) had a 5-bp sequence (TATAT) [17,27] (Figure 2C). The AT content of the surrounding 6-bp sequence was significantly greater than that of the distal sequence, with the highest AT content observed at TSD loci t1, t2, and t3. The symmetrical distribution of the AT (or GC) content is consistent with the transposition mechanism of transposons Tn*1721* and Tn*7052* between plasmids [28,29]. The transition from GC to AT reflects the interaction of transposons with insertion DNA sites, which may be crucial for distorting or bending DNA to facilitate accessibility of the transposon terminus [30].

To analyze whether the position of the P1 phage carrying ARG was biased, we searched for phage transporting drug resistance genes in the NCBI database and analyzed the positions of the drug resistance genes. A total of 317 P1 phage genomes were obtained from the NCBI database, and 14 P1 phage genomes carrying ARGs were screened through the Center for Genomic Epidemiology (CGE, http://www.genomicepidemiology.org/, accessed on 30 April 2023) ARG search (Supplemantary Materials Table S1). Among the 14 P1 phage-carrying ARGs, 10 were *E. coli* P1 phages, three were *Salmonella* P1 phages and one was a *K. pneumoniae* P1 phage. Three of the *Salmonella* P1 phages carried the same transposon, with a size of approximately 104 kb, and the remaining 11 were P1 phage-plasmids which merged with the ARG-carrying plasmids through different mobile elements; in addition, the phage-plasmid size ranged from approximately 100 to 300 kb. The locations of phage P1 carrying ARGs or fusion with plasmids are as follows: *ref* was involved in recombination enhancement; *mat* and *lxr* were involved in particle maturation; *ant1*, and *pacA* were involved in putative morphogenetic function or SOS putative morphogenetic function; *upfA* and *upfB* were involved in plasmid replication; and *cin*, *gp21* and *gpU* were involved in encoding holing and tail fiber-related proteins.

Wang’s study revealed the similar structural characteristics and gene content of 77 P1-like PPs in the RefSeq database. The P1 phage contained eight highly variable regions with low GC content, which suggested that it is a hot spot for ARG mobilization [13]. On the other hand, all the ARG genes or plasmids that bind to phages are inserted sequences or transposon genes, including IS*1380*, IS*Kpn19*, IS*Apl1*, IS*421*, IS*6*, IS*Ec45*, IS*26*, Tn*1721*, and Tn*3*. Studies have shown that mobile genetic elements also have low GC values, which might help the insertion sequence fuse with highly variable regions in the phage.

Generalized transduction is when phages mispackage the host genome for gene exchange; however, in some cases, the phage particles that package the host genes are transmitted to a new host and lose the ability to be transmitted further [31]. A limited range of host gene packing is used to carry out specific transduction, which is accomplished by packaging sections of the host gene next to the phage. Recent discoveries have shown that lateral transduction is significantly more effective at transducing host genes than at transducing conjugation elements [11]. The P1 phage is a generalized transduction phage that exists as a free plasmid. According to our research, an ARG-carrying P1 phage can generate and transduce ARGs.

## 3. Materials and Methods

### 3.1. Plaque Assays and TEM Assays

*Salmonella* J46, which carried P1-CTX, was selected as the induction target. Analysis on PHARSTER (http://phaster.ca/, accessed on 31 December 2023) showed that J46 contained six prophages, of which only two were complete phages, including P1-CTX and Salmon_SEN8_NC_047753. To assess the lytic capacity of the P1-CTX phage, we conducted a plaque assay following a modified method described by Wang et al. [32]. Log phase cultures *Salmonella* J46 with an optical density at 600 nm (OD_600_) value of 0.5 were induced using MitC (2 mg/L). The culture was then filtered through a 0.22 μm filter (Millipore). Dilutions of the P1-CTX phage were prepared in a sterile SM buffer (10 mM NaCl, 10 mM MgSO_4_, 50 mM Tris·HCl, pH 7.5). Subsequently, 100 μL volumes of exponential-phase cultures of *Salmonella* BYC4 were incubated with the diluted P1-CTX phage for ten minutes at 30 °C. After incubation, samples were mixed with Luria−Bertani soft agar (0.7% agar) on LB plates and incubated overnight at 37 °C to determine plaque formation. The clear plaques formed on the top agar layer were selected and added to SM buffer, totaling up to 500 μL. Following vortexing, the culture was centrifuged at 10,000× *g* for 10 min. Then, the supernatant was filtered through a disposable sterile syringe filter and DNase I was added to filtrate to eliminate bacterial genomic DNA. The P1-CTX phage was detected by PCR, and the filtrate was stored at 4 °C until use.

Phage morphology was examined as described by Wang et al. [32]. The purified high-titer stock P1-CTX phages were absorbed on copper grids. Drops were then blotted and negatively stained with phosphor-tungstic acid (PTA, 1%, pH = 7), and samples were examined by TEM (FEI/Talos F200S).

### 3.2. Induction Conditions

Ten antibiotics (ciprofloxacin, gentamicin, amikacin, colistin, meropenem, trimethoprim-sulfamethoxazole, chloramphenicol, kanamycin, fosfomycin, cefotaxime), ultraviolet (UV) radiation, and a temperature of 42 °C were selected for inducing, respectively. Each antibiotic was added to log phase cultures of *Salmonella* J46. Samples were incubated for 4 h at 37 °C with gentle shaking, then filtered through a 0.22 μm filter. The concentrations of antibiotics selected in this study are shown in Table S2.

The impact of the UV radiation light-induced phage and 42 °C were tested by a similar method. An amount of 1 mL of the LB broth log phase cultures of *Salmonella* J46 was pipetted into a sterile Petri dish and then placed under the UV radiation light source with the lid off for 30 s. Exposed bacteria were filtered. An amount of 1 mL of the LB broth log phase cultures of *Salmonella* J46 was placed in a 42 °C water bath for 5 min and then filtered, while 37 °C was used as the control condition. Dilutions of the P1-CTX phage were made in a sterile SM buffer. A volume of 100 μL of exponential-phase cultures of *Salmonella* BYC4 was incubated with 100 μL of diluted P1-CTX phage at 30 °C for 10 min. An amount of 10 mL of LB soft agar was added for double-layer plates and incubated overnight.

### 3.3. Transduction and Host

Transduction was performed as described previously [19]. *E. coli* K12 and DH5α, *Salmonella* ATCC14028 and SL1344, and 200 clinically isolated *E. coli* and *Salmonella* were used for recipients. Recipient cells in the log phase were mixed with 1 mL phage P1-CTX, and CaCl_2_ was then added to a final concentration of 10 mM. Samples were incubated at 30 °C for 30 min. After incubation, 1 M sodium citrate was added to a final concentration of 15 mM and then spread on LB agar plates supplemented with 2 mg/L cefotaxime and incubated at 37 °C overnight. The presence of *bla*_CTX-M-27_ in transductant was further confirmed by PCR and sequencing. All transductants were subjected to S1-PFGE and Southern blotting using digoxigenin-labeled probes specific for the *bla*_CTX-M-9G_-like gene, as described previously [17].

The frequencies of transduction were represented as the number of colonies on the cefotaxim selective LB plates per the initial number of colonies on the non-selective LB plates (colony-forming unit; CFU) of the recipient or initial plaque-forming unit (PFU) of the phage. To ensure accuracy and reliability, three independent transduction experiments were conducted and their results were averaged.

### 3.4. Whole-Genome Sequencing

Recipients and transductants were selected for long-read sequencing (Oxford Nanopore, Oxford, UK). De novo hybrid assembly using short and long reads was performed using Unicycler v0.4.4 [33,34]. Sequencing quality and statistics per isolates were checked using the QualiMap v2.2.2 [35]. The P1 phage sequences were annotated using RAST (http://rast.nmpdr.org/rast.cgi, accessed on 31 December 2022) and BLAST (http://blast.ncbi.nlm.nih.gov/Blast.cgi, accessed on 31 December 2022) [36]. ARGs were identified using ResFinder (https://cge.cbs.dtu.dk/services/ResFinder/, accessed on 31 December 2022) [37]. Mobile elements were identified using ISfinder (https://www-is.biotoul.fr/, accessed on 31 December 2022) [38].

### 3.5. ARGs in P1 phage

For evaluating the location for acquiring ARGs on P1-like phages, phage P1-carrying ARGs were searched in the NCBI database (https://www.ncbi.nlm.nih.gov/, accessed on 30 April 2023). The insertion sites of ARGs were analyzed using ResFinder, and the plasmid replicon types were identified using PlasmidFinder (https://cge.cbs.dtu.dk/services/PlasmidFinder/, accessed on 30 April 2023). The motif sequence logo was drawn using WebLogo [39].

## 4. Conclusions

In this study, we found that the P1-CTX phage can be induced under different conditions, lyse the host, and be transduced into *E. coli* and a variety of serotypes of *Salmonella*. The *bla*_CTX-M-27_ gene acquisition mechanism of the P1 phage showed Tn*1721* movement of *bla*_CTX-M-27_ between the P1 phage gene *ref* and *mat*. Moreover, the mechanism underlying the P1 phage acquisition of ARGs may involve insertion sequences or transposons as carriages move into phage variable regions. 

## Figures and Tables

**Table 1:** Information on bacterial hosts that were successful recipients of P1-CTX phage transduction.

Strain	Species	Serotype	Source	Isolate Date
G67, H13, H35, H45, H75	*Salmonella*	Derby	Pork	2021
R17 A85 B9 A92, B25		Rissen	Pork	2015 2014 2013 2016
BYC5, BYC8 YM7, YM11, JZ102		Kentucky	Chicken meat Pork	2016
G5, H5, H9, H43		Typhimurium	Pork	2021
BYC4 YXC6 H49		Cerro	Chicken meat Chicken meat Pork	2016 2016 2021
LJ23		Senfutenber	Duck	2009
HYM2		Menster	Duck	2018
RC15, HJM51 H145	*Escherichia coli*	ND	Chicken Pork	2018 2021

ND: Not determined.

## Data Availability

The HYM1 and T-HYM1 in this study were deposited in the NCBI database. The complete nucleotide sequence of p1-HYM1 and p1-T-HYM1 were deposited in GenBank under the accession numbers CP141766 and CP141768, respectively.

## Supplementary Materials

The following supporting information can be downloaded at: https://www.mdpi.com/article/10.3390/antibiotics13050446/s1, Table S1: Characteristics of antibiotic resistance gene in P1 phage genome. Table S2: The minimal inhibitory concentration of 10 antibiotics of *Salmonella* J46.
