# Supplementary material for: Characterization of Salmonella Phage P1-CTX and the Potential Mechanism Underlying the Acquisition of the blaCTX-M-27 Gene
Source: Antibiotics (Basel). 2024 May 14;13(5):446. doi: 10.3390/antibiotics13050446 (PMC11117986; doi:10.3390/antibiotics13050446)
Supplement: Supplementary file 1 [file antibiotics-13-00446-s001.zip › Table S1.pdf]

Table S1 Characteristics of P1 phage carrying antibiotic resistance gene

| Name | GenBank No. | Strain | Location | Source | Size（kb） | Antibiotic resistance gene | Plasmid type | Mobile elements connect with P1 phage | Gene loci in P1 phage |
|---|---|---|---|---|---|---|---|---|---|
| SJ46 | KU760857 | *Salmonella* | China | Pork | 103.4 | *bla*$_{\text{CTX-M-27}}$ | N/T | Tn*1721* | *ref*, *mat* |
| unnamed2 | CP031191 | *Salmonella* | China | Chicken | 103.4 | *bla*$_{\text{CTX-M-27}}$ | N/T | Tn*1721* | *ref*, *mat* |
| p14146 | CP064673 | *Salmonella* | China | Chicken meat | 103.4 | *bla*$_{\text{CTX-M-27}}$ | N/T | Tn*1721* | *ref*, *mat* |
| p14076B | CP064676 | *Salmonella* | China | Chicken meat | 103.4 | *bla*$_{\text{CTX-M-27}}$ | N/T | Tn*1721* | *ref*, *mat* |
| JL22 | ON018986 | *Escherichia coli* | China | Duck | 99.6 | *bla*$_{\text{CTX-M-55}}$ | N/T | IS*1380* | *upfB* |
| unnamed3 | CP041922 | *Escherichia coli* | China | Sputum | 94.6 | *bla*$_{\text{CTX-M-55}}$ | N/T | IS*1380* | *upfA* |
| pEC5502-2 | CP092645 | *Escherichia coli* | China | Urine | 99.2 | *bla*$_{\text{CTX-M-55}}$ | N/T | IS*Kpn19*, Tn*3* | *cin, lxr* |
| pSCEC128 | MH844525 | *Escherichia coli* | China | - | 101.1 | *mcr-1* | N/T | IS*Apl1* | *ant1* |
| p1-HYM1 | CP141766 | *Escherichia coli* | China | Duck | 96.1 | *mcr-1* | N/T | IS*Apl1* | *ant1* |
| pTB-nb1 | CP033632 | *Escherichia coli* | China | Chicken | 147.4 | *sul1*, *aadA1*, *dfrA1*, *aph(6')-Id*, *aph(3")-Ib*, *sul2*, *aac(3)-IId*, *aph(3')-IIa*, *oqxAB*, *bla*$_{\text{CTX-M-65}}$, *fosA3*, *tet(A)* | IncHI2 | IS*421*, IS*6* | *gp21, phage protein* |
| pMB3176_1 | CP103719 | *Escherichia coli* | USA | Blood | 250.7 | *sul1*, *aadA5*, *dfrA17*, *bla*$_{\text{CTX-M-15}}$, *bla*$_{\text{OXA-1}}$, *aac(6')-Ib-cr5*, *tetR(B)*, *tet(B)*, *tet(C)* | IncF | IS*Ec45* | *upfB* |
| pTZ20_1P | MN510447 | *Escherichia* | Australia | Pig | 130.1 | *dfrA12*, *aadA2*, *cmlA*, *aadA1*, *sul3*, *bla*$_{\text{TEM-}}$ | IncF | IS*26*, Tn*1721* | *cin, pacA* |

| | | | | | | | | | |
|---|---|---|---|---|---|---|---|---|---|
| | | *coli* | | | | $_{1B}$, *aac(3)-IV*, *aph(4)-Ia* | | | |
| pFUJ80155-1 DNA | AP024695 | *Escherichia coli* | Japan | Human | 147.0 | *bla*$_{TEM-1A}$, *sul3*, *cmlA1*, *tetX*, *aadA1*, *aadA2*, *dfrA12* | IncFIB | Tn*3* | *ref* |
| pCRKP-59-KPC | KX928752 | *Klebsiella pneumoniae* | China | Patient | 216.9 | *qepA1*, *rmtB1*, *bla*$_{TEM-1}$, *aac(3)-IId*, *bla*$_{KPC-}$ $_{2}$, | IncF | IS*6* | *gpU* |

N/T: not typeable; -: no information;
