# Supplementary material for: Characterization of Salmonella Phage P1-CTX and the Potential Mechanism Underlying the Acquisition of the blaCTX-M-27 Gene
Source: Antibiotics (Basel). 2024 May 14;13(5):446. doi: 10.3390/antibiotics13050446 (PMC11117986; doi:10.3390/antibiotics13050446)
Supplement: Supplementary file 1 [file antibiotics-13-00446-s001.zip › Table S2.pdf]

Table S2 The minimal inhibitory concentration (MIC) of 10 antibiotics of *Salmonella* J46

Antibiotics	MIC(mg/L)	½ MIC(mg/L)
ciprofloxacin	32	16
gentamicin	1	0.5
amikacin	8	4
colistin	1	0.5
meropenem	2	1
trimethoprim-sulfamethoxazole	32	16
chloramphenicol	32	16
kanamycin	1024	512
fosfomycin	1	0.5
cefotaxime	128	64
